# Biological Properties of IRIM, the Iridium(III) Analogue of (Imidazolium (Bisimidazole) Tetrachlororuthenate) (ICR)

**DOI:** 10.1155/MBD.2000.195

**Published:** 2000

**Authors:** G. Marcon, A. Casini, P. Mura, L. Messori, A. Bergamo, P. Orioli

**Affiliations:** 1 CIRCMSB Unit of Florence via Gino Capponi 7 Florence I-50121 Italy; 2 Department of Chemistry University of Florence Florence I-50121 Italy; 3 Istituto di Strutturistica Chimica “G. Gia6omello” Area della Ricerca di Roma CNR CP10 Monterotondo Stazione, Rome I-00016 Italy; 4 Fondazione Callerio via A. Fleming 22 Trieste 31 I-34127 Italy

## Abstract

Some biological aspects of the new complex imidazolium bisimidazole tetrachloro iridate(III)-IRIM- the iridium(III) analogue of ICR, were considered. More in detail the conformational effects produced by IRIM on DNA and the cytotoxic properties of IRIM on some selected human cell lines were measured. Dialysis experiments and DNA thermal denaturation studies are suggestive of poor binding of IRIM to DNA; formation of interstrand crosslinks is not observed. In any case CD measurements suggest that addition of increasing amounts of IRIM to calf thymus DNA results into significant spectral changes, that are diagnostic of a direct interaction with DNA. A number of experiments carried out on the A2780 human ovarian carcinoma, B16 murine melanoma, MCF7 and TS mammary adenocarcinoma tumor cell lines strongly point out that IRIM does not exhibit significant growth inhibition effects within the concentration range 10^-4^-10^-6^ M. It is suggested that the lower biological effects of IRIM compared to ICR are a consequence of the larger kinetic inertness of the iridium(III) center with respect to ruthenium(III).


### BIOLOGICAL PROPERTIES OF IRIM, THE IRIDIUM(III) ANALOGUE OF (IMIDAZOLIUM (BISIMIDAZOLE) TETRACHLORORUTHENATE) (ICR)

G. Marcon[1], A. Casini[2], P. Mura[3], L. Messori[2], A. Bergamo[4] and P. Orioli*[2]

[1] CIRCMSB, Unit of Florence, via Gino Capponi 7, I-50121 Florence, Italy

[2] Department of Chemistry, University of Florence, I-50121 Florence, Italy

<orioli@cerm.unifi.it>

[3] Istituto di Strutturistica Chimica "G. Giacomello" Area della Ricerca di Roma, CNR CP10, I-00016 Monterotondo Stazione, Rome

[4] Fondazione Callerio, via A. Fleming 22,31 I-34127 Trieste, Italy

### ABSTRACT

Some biological aspects of the new complex imidazolium bisimidazole tetrachloro iridate(III) -IRIM- the iridium(III) analogue of ICR, were considered. More in detail the conformational effects produced by IRIM on DNA and the cytotoxic properties of IRIM on some selected human cell lines were measured. Dialysis experiments and DNA thermal denaturation studies are suggestive of poor binding of IRIM to DNA; formation of interstrand crosslinks is not observed. In any case CD measurements suggest that addition of increasing amounts of IRIM to calf thymus DNA results into significant spectral changes, that are diagnostic of a direct interaction with DNA. A number of experiments carried out on the A2780 human ovarian carcinoma, B16 murine melanoma, MCF7 and TS mammary adenocarcinoma tumor cell lines strongly point out that IRIM does not exhibit significant growth inhibition effects within the concentration range $10^{-4}$-$10^{-6}$ M. It is suggested that the lower biological effects of IRIM compared to ICR are a consequence of the larger kinetic inertness of the iridium(III) center with respect to ruthenium(III).

### INTRODUCTION

Following the success of cisplatin as an anticancer agent, new interest raised on the design and development of new metal based antitumor agents [1,2]. Within this frame Keppler and coworkers prepared and characterized a series of antitumor ruthenium(III) complexes, of which ICR (imidazolium bisimidazole tetrachloro ruthenate(III)) is probably the most representative [1]. Later on, Alessio and Sava developed NAMI A, a compound structurally related to ICR, that has recently entered phase I clinical trials [3]. The biological evaluation of these innovative metal complexes has clearly pointed out that the mechanism of action of ruthenium compounds is substantially different from that of platinum complexes. Generally the direct cytotoxic effects of ruthenium compounds are much lower than those produced by equimolar amounts of cisplatin while the antimetastatic effects are more pronounced [3]. Also the direct DNA damaging effects are of much lower intensity [4]. In this context it is of interest to evaluate the biological effects of isostructural metal complexes, obtained by simple replacement of the central metal ion. The comparative analysis of the pharmacological profiles provides information on the specific role of the metal. Ruthenium(III) complexes may be easily mimicked by rhodium(III) complexes and iridium(III) complexes. Some studies already appeared on the rhodium(III) analogue of ICR [5]; the iridium(III) analogue of ICR -IRIM- has been recently synthesized by Mura [6].

**IRIM**

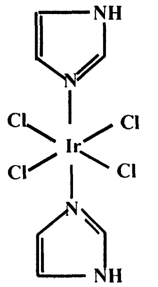

**Figure 1** Schematic drawing of the imidazolium bisimidazoletetrachloroiridate(III)-IRIM-complex.

The most striking difference between ICR and its rhodium and iridium analogues consists in the increasing kinetic inertness of the metal center. In this study we report on some significant biological properties of IRIM. More specifically the effects of IRIM on DNA stability and conformation in solution and its cytotoxic properties toward a number of human tumor cell lines were investigated.

### MATERIALS AND METHODS

IRIM was synthetized as reported [6]. The purity of the compound was checked through elemental analysis and $^{1}H$ NMR spectroscopy. *Calf thymus* DNA was purchased from Sigma Chemical Company. All other reagents used in this study were of analytical grade and were commercially available.

Electronic absorption spectra were measured with a Perkin Elmer Lambda 20 Bio instrument.

Electronic spectra of IRIM $3.6x10^{-4}$ M were recorded before and after addition of *calf thymus* DNA (r= 0.7) in the phosphate 50 mM, NaCl 4 mM, pH 7.4 buffer and the same sample was dialyzed after 24h incubation. The spectra of the upper portion of the solution containing *calf thymus* DNA and of the lower portion of the solution, containg only the iridium(III) complex, were recorded.

All melting measurements were carried out in the $10^{-2}$ M $NaClO_4$ and $10^{-3}$ M NaCl buffer. *Calf thymus* DNA was dissolved in the buffer and DNA concentration determined by absorption measurements at 260 nm. DNA concentration was equal to $3.6x10^{-5}$ M [nucleotide]. DNA was treated with IRIM at different mol/bp ratios (r= 0.01, 0.1, 1.0) and each sample was incubated for 24 hrs at room temperature.

Thermal denaturation experiments were performed in quartz cuvettes with a Perkin Elmer Lamba 20 Bio spectrophotometer equipped with a thermostated cell as described in reference. Samples were continuously heated with a rate of temperature increase of 0.5°C/min while monitoring the absorbance changes at 260 nm. The investigated interval of temperature ranged from 50°C to 90°C. Upon reaching 90°C, samples were cooled back to 40°C in order to follow the renaturation process. Values for melting temperatures ($T_m$) and for the melting interval ($\Delta T$) were determined according to the reported procedures [7].

CD spectra were recorded at increasing IRIM/*calf thymus* DNA ratio (r= 0.0, 0.01, 0.1, 1.0) after mixing on a Jasco J600 dichrograph operating at room temperature, interfaced with a PC, and analyzed through the standard Jasco software package.

Cytotoxicity measurements were carried out on the A2780 ovarian carcinoma cell line, both sensitive and resistant to cisplatin (A2780/S and A2780/R) following the Sulphorhodamine-B (SRB) procedure [8], and data analyzed through standard methods. Cells were exposed to the cytotoxic agent for 72 hours.

The activity of IRIM on the B16 melanoma, MCF7 breast cancer and TS adenocarcinoma tumor cell lines was also analyzed, upon treatment 24 or 72 hours long. Cell growth of samples treated with increasing concentrations of IRIM $-10^{-6}$M, $10^{-5}$M and $10^{-4}$M – was monitored in comparison to controls through the microculture tetrazolium test (MTT) [9] or SRB method [8].

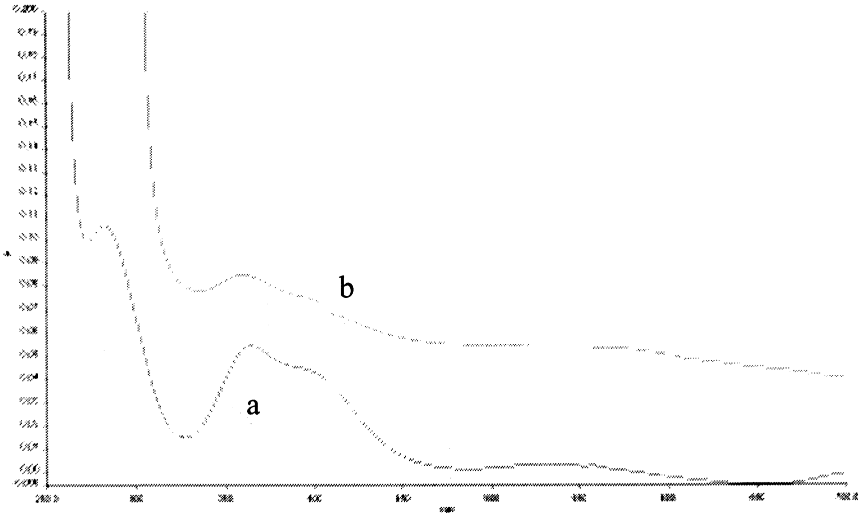

**Figure 2** Electronic spectra of IRIM (a) $3.6x10^{-4}$ M following addition of *calf thymus* DNA r= 0.7 (b) in phosphate buffer 50 mM, NaCl 4 mM, pH 7.4.

### RESULTS

*Interactions of IRIM with calf thymus DNA*

The interactions of IRIM with *calf thymus* DNA *in vitro* was first analyzed by visible spectroscopy. As previously reported [6], IRIM is soluble in a physiological buffer and gives rise to a characteristic absorption spectrum in the visible. Notably, the visible spectrum of IRIM does not change over a period of several hours, at room temperature, implying that the metal chromophore is relatively stable under physiological conditions [6]. In order to monitor the reaction of IRIM with DNA, the visible spectra of IRIM were recorded following addition of saturating amounts of *calf thymus* DNA. Notably the presence of DNA does not affect significantly the visible spectrum of IRIM implying that the metal chromophore is substantially conserved.

On the other hand ultradialysis experiments are suggestive of weak binding of IRIM to *calf thymus* DNA: either the fraction containing *calf thymus* DNA or the DNA-free fraction contain comparable amounts of IRIM following ultrafiltration.

The effects that IRIM produces on the structure and conformation of *calf thymus* DNA were further investigated through circular dichroism and DNA melting studies. The CD technique is indeed extremely sensitive to the conformation of DNA double helix. CD spectra of *calf thymus* DNA upon addition of increasing amounts of IRIM are shown in **Figure 3**.

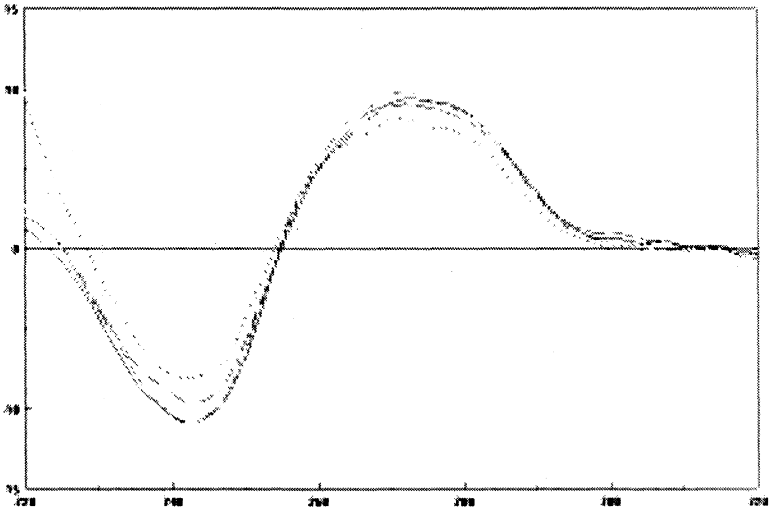

**Figure 3** Circular dichroism spectra of *calf thymus* DNA upon addition of increasing amounts of IRIM. Spectra were recorded at the following r values: 0.0, 0.01, 0.1 and 1.0 (from top to bottom at 260 nm). Conditions: phosphate 50 mM, NaCl 4 mM, pH 7.4 buffer, *calf thymus* DNA $3.6 \times 10^{-5}$ M (basepair concentration).

It is observed that IRIM affects somehow the CD spectrum of DNA. The effects are of larger intensity as the ratio IRIM/*calf thymus* DNA is increased from r=0.01 to 0.1 and to 1. A progressive decrease of the positive band at 275 nm and of the negative one at 240 nm is observed, with an isosbestic point at 260 nm. This observation suggests that addition of increasing amounts of IRIM induces a conformational transition of *calf thymus* DNA within the frame of a B-type conformation.

Thermal denaturation profiles of *calf thymus* DNA were investigated in the presence of increasing amounts of IRIM: the same pattern of IRIM concentrations of the CD studies was investigated (r=0.01, 0.1 and 1.0). A representative complete melting profile (heating and cooling process) is shown in **Figure 4** and the relevant parameters extracted from the individual experiments are reported in **Table 1**.

**Table 1** Experimental $T_m$ (°C), ΔT (°C) and ΔH values as determined from the thermal denaturation profiles of *calf thymus* DNA and of the adducts IRIM/*calf thymus* DNA at the ratios 0.01, 0.1 and 1.0; data on the adducts ICR/*calf thymus* DNA (r=0.01 and 0.1) are shown.

| | $T_m$ (°C) | ΔT (°C) |
|---|---|---|
| ***Calf thymus* DNA** | 67.63 | 10.80 |
| **IRIM/ctDNA r=0.01** | 67.05 | 9.98 |
| **IRIM/ctDNA r=0.1** | 67.50 | 9.88 |
| **IRIM/ctDNA r=1** | 67.00 | 11.43 |
| **ICR/ctDNA r=0.01** | 66.70 | 11.60 |
| **ICR/ctDNA r=0.1** | 67.50 | 9.90 |

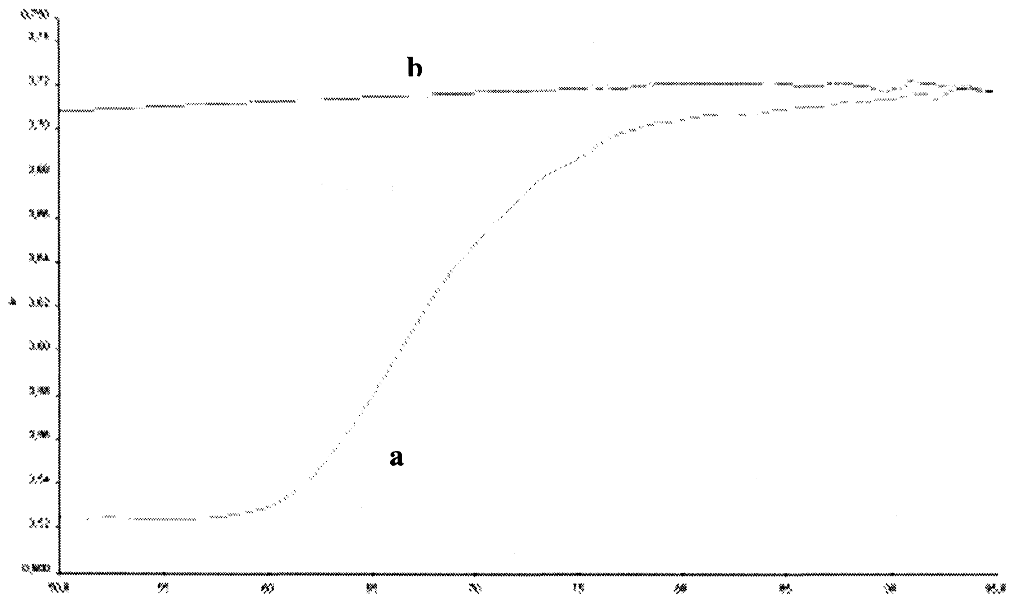

**Figure 4** Denaturation (a) and "renaturation" (b) profiles of *calf thymus* DNA 3.6x$10^{-5}$ M as collected by melting measurements.

It clearly emerges that addition of increasing amounts of IRIM does not affect significantly the melting temperature of *calf thymus* DNA. In other word there is no evidence of stabilization or destabilization of the double helix following IRIM addition. It can just be observed that at the highest ratios the $\Delta T$ value is slightly increased. The DNA renaturation profiles show that the presence of IRIM does not favor at all the strand pairing process after melting, ruling out that IRIM is able to induce formation of interstrand crosslinks.

*Cytotoxic effects of IRIM*

The cytotoxic properties of IRIM in vitro were evaluated through two distinct experiments. They were first analyzed on the A2780 ovarian carcinoma cell line either sensitive or resistant to cisplatin in comparison to ICR and CDDP. Results are shown in **Table 2.**

It clearly emerges that IRIM produces a very modest growth inhibition effect; $IC_{50}$ values larger than 150 μM were estimated on the cisplatin sensitive line to be compared with values of 80 μM for ICR and 2 μM for cisplatin. Far larger $IC_{50}$ values for all complexes, within the same order of cytotoxic potency, were found in the case of the cisplatin resistant line A2780/R.

**Table 2** $IC_{50}$ values (μM) of cisplatin-sensitive and -resistant human tumor cell lines [A2780/S and A2780/R of ovarian carcinoma] towards IRIM and ICR. For comparison purposes the values obtained with CDDP are reported. The values reported in this table are the average± SE of 3 experiments for IRIM, the average of 2 experiments for ICR and the average± SE of 5 experiments for CDDP.

| | **A2780/S** | **exp. n.** | **A2780/R** | **exp. n.** |
|---|---|---|---|---|
| **IRIM** | 167.5±46.9 | 3 | >480 | 3 |
| **ICR** | 80 | 2 | >240 | 2 |
| **CDDP** | 1.6±0.58 | 5 | 16.1±3.85 | 5 |

Later on, the cytotoxic effects of IRIM were monitored on the following human tumor cell lines: B16 melanoma, MCF7 breast cancer and TS adenocarcinoma. Cell growth of samples treated with increasing concentrations of IRIM – $10^{-6}$M, $10^{-5}$M and $10^{-4}$M – was monitored in comparison to controls through the MTT or SRB methods. Again no significant difference was detected among treated samples and controls, pointing out that the inhibitory effects of IRIM are indeed very poor.

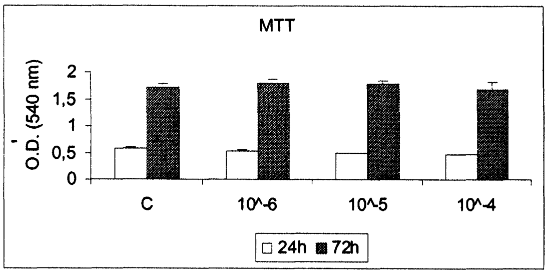

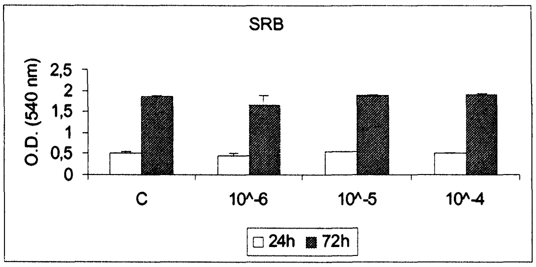

B16-F10 cellular line

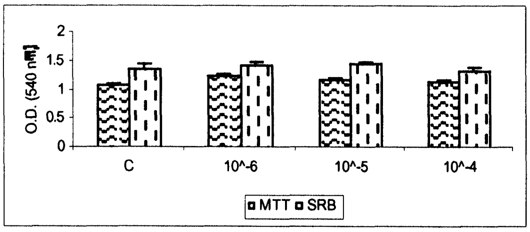

MCF-7 cellular line (after 24 hours treatment)

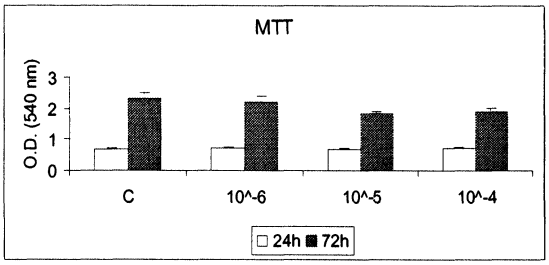

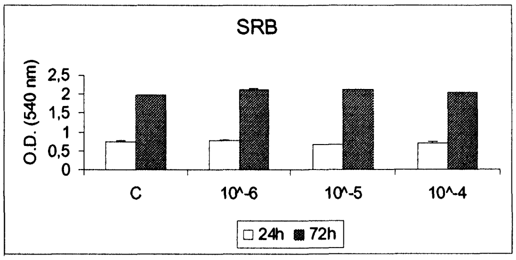

TS/A cellular line

**Figure 5** Cytotoxic activity of IRIM towards the B16 murine melanoma cell line, MCF7 and TS mammary adenocarcinoma cell lines (after 24 or 72 hours treatment). IRIM had three different concentrations ($10^{-6}$, $10^{-5}$ e $10^{-4}$M); vitality was measured following the MTT and SRB procedures. The values reported in this graphs are the average± SE of 3 experiments.

### DISCUSSION

Metal complexes other than platinum are today actively investigated as potential cytotostatic and antitumor drugs. In this frame, much work has been devoted to the analysis of ruthenium(III) complexes that showed in some cases promising biological and pharmacological profiles. A ruthenium(III) complex- NAMI A- is presently undergoing phase I clinical trials. Structural analogues of ruthenium(III) complexes may be prepared by using rhodium(III) or iridium(III) as the central metal ion. We are exploiting iridium(III) analogues of known antitumor ruthenium(III) complexes as novel antitumor agents; the aim is to define the chemical and biological consequences resulting from the replacement of the ruthenium(III) center with the more inert iridium(III) center.

In this paper we report on the biological behaviour of IRIM, trans bisimidazole tetrachloro iridate(III), compared to ICR. Specifically two aspects of the pharmacological behaviour of IRIM have been stressed: i) the interaction with DNA; ii) the cytotoxic effects on a number of human tumor cell lines.

*i) The interaction with DNA*

DNA was selected since it represents a major target for platinum(II) antitumor compounds and is the probable target for several other antitumor metal complexes. The interaction of IRIM with *calf thymus* DNA was monitored through a number of classical screening techniques including circular dichroism, analysis of DNA thermal denaturation and dialysis experiments. This approach permits to establish whether i) the metal chromophore undergoes transformations upon binding; ii) the conformation of DNA is modified; iii) the double helix is stabilized, destabilized or unaffected; iv) interstrand crosslinks are formed.

Overall, IRIM produces modest effects on DNA in the sense that the metal chromophore is virtually unaffected by addition of DNA; the stability of the double helix is only weakly decreased even at the highest ratios; formation of interstrand crosslinks is not observed. Some evidence of interaction is obtained from CD spectra indicating the occurrence of slight conformational changes of the DNA solution structure. A possible interpretation of these results stems on the fact that IRIM is far less reactive than ICR since ligand exchange reactions are much slower. In the case of ruthenium(III) complexes like ICR and NAMI, it is assumed that reactions with biologically relevant targets take place following chloride hydrolysis. It is straightforward to hypothesize that IRIM does not form coordinative bonds with DNA since it is not able to hydrolyze to a significant extent under physiologically occuring conditions. The only type of reactivity with DNA that can take place in the absence of hydrolysis is some groove interaction. In the light of the present results, it can be stated that IRIM exhibits very modest DNA modifying properties.

*ii) The cytotoxic properties.*

The lack of DNA modifying properties does not necessarily imply that IRIM must be discarded a priori from any further pharmacological evaluation. Indeed, apart from DNA binding, other mechanisms can be responsible for the antitumor activity, such as binding to proteins involved in DNA methabolism or implicated in malignant transformation and maintenance of the transformed phenotype. With this in mind, we further tested the biological properties of IRIM by analyzing its cytotoxic properties toward selected human tumor cell lines. Four major lines were considered, namely A2780 ovarian carcinoma, B16 melanoma, MCF7 breast cancer and TS adenocarcinoma tumor cell lines. We found that IRIM shows very modest cell growth inhibition effects. The comparison with cisplatin is striking: on the A2780 line the $IC_{50}$ value of cisplatin is 2 μM, that of IRIM is 140 μM; nevertheless, it is worthwhile noting that the $IC_{50}$ value of ICR on the same cell line is 80 μM, close to that of IRIM. Indeed the cytotoxicity test has been longly considered and it must be stressed that many compounds with good tumor cell-killing activity have been discovered, but few have found clinical utility. On the other side, in recent years, several non cytotoxic compounds – among which some ruthenium(III) complexes - turned out to exhibit relevant anticancer properties. Thus, we must approach the cytotoxicity test considering the following limits: 1) a lack of discrimination between effects on tumor and normal tissue, 2) the cell-cycle dependency of many cytotoxic drugs and 3) the frequent susceptibility of these substances to induced drug resistance. To conclude, in our opinion, before stating that IRIM and similar iridium(III) complexes are not useful as antitumor agents, it is necessary to perform more advanced in vitro and in vivo pharmacological studies.

### CONCLUSIONS

*In vitro* interactions of IRIM with *calf thymus* DNA were analyzed through various physicochemical techniques. It is found that IRIM induces small conformational changes on DNA. Overall the observed effects are much weaker than those induced by cisplatin. Notably DNA modifications brought about by ICR are larger than those produced by IRIM. It is hypothesized that the interaction of IRIM with DNA is essentially non-covalent in nature whereas ICR is able to form coordinative bonds. The weak DNA modifying properties correlate with low cytotoxicity on a number of human tumor cell lines.

### ACKNOWLEDGMENTS
Dr. S. Carotti (Laboratory of Chemoterapy, Pharmacology, University of Florence) is acknowledged for helping us with the cytotoxicity tests.

### REFERENCES

1. *Metal Complexes in Cancer Chemotherapy* Keppler, B. K. (ed.) Weinheim: VCH, 1993;
2. *Medicinal Inorganic Chemistry* in *Chem. Rev.*, 1999, 99 (9), 2201-2842;
3. Sava, G., Alessio, E., Bergamo, A., Mestroni, G. *Topics BioInorg. Chem.* 1999, 1, 143-169;
4. Gallori, E., Vettori, C., Alessio, E., Gonzalez, F., Vilchez, Vilaplana, R., Orioli, P., Messori, L. and Casini, A. *Arch. Biochem Biophys.* 2000, 376 (1), 156-162;
5. Mestroni, G., Alessio, E., Santi, A.S., Geremia, S., Bergamo, A., Sava, G., Boccarelli, A., Schettino, A., Coluccia, M. *Inorg. Chim. Acta* 1998, 273, (1-2), 62-71,
6. Mura, P.. Casini, A., Marcon, G. and Messori, L. *Inorg. Chim. Acta,* 2000, submitted;
7. Wilson WD, Tanios F.A., Fernandez-Saiz M, and Rigl CT in *Methods in Molecular Biology,* Fox KR editor, Humana Press NJ, 1997, 90;
8. Skehan, P., Storeng, R., Scudiero, D., Monks, A., Mcmahon, J., Vistica, D., Warren, J.T., Bokesch, H., Kenney, S. and Boyd, M.R. *J. Natl. Cancer Inst.* 1990, 82 (13), 1107-1112;
9. Mosmann, T. *J. Immunol. Methods* 1983, 65 (1-2), 55-63.

**Received: June 23, 2000 - Accepted: August 22, 2000 -**
**Received in revised camera-ready format: September 21, 2000**

